# An HPLC and UHPLC-HRMS approach to study PSMA-11 instability in aqueous solution

**DOI:** 10.1186/s41181-021-00122-3

**Published:** 2021-03-24

**Authors:** Antonella Iudicello, Filippo Genovese, Valentina Di Iorio, Gianfranco Cicoria, Stefano Boschi

**Affiliations:** 1 Pharmaceutical Department, Azienda USL of Modena, Largo del Pozzo, 71, 41121 Modena, Italy; 2 Oncology and Hematology Department, Nuclear Medicine Unit, Azienda Ospedaliero-Universitaria of Modena, Largo del Pozzo, 71, 41121 Modena, Italy; 3 Centro Interdipartimentale Grandi Strumenti, University of Modena and Reggio Emilia, Via Campi 213/A, 41125 Modena, Italy; 4 Istituto Scientifico Romagnolo per lo Studio e la Cura dei Tumori (IRST) – IRCCS IRST, Via Piero Maroncelli, 40, 47014 Meldola, FC Italy; 5 Medical Physics Department, Azienda Ospedaliero-Universitaria of Modena, Largo del Pozzo, 71, 41121 Modena, Italy; 6 Department for Life Quality Studies, University of Bologna, Corso D’Augusto, 237, Rimini, 47921 Italy

**Keywords:** [^68^Ga]Ga-PSMA-11, PSMA-11, Quality control, Radiopharmaceuticals, Reagents instability, Reference solution

## Abstract

**Background:**

The stability of precursors and reagents is of utmost importance for developing a robust radiolabelling method that provides high and constant radiochemical yield and radiochemical purity.

While performing the QC of the [^68^Ga]Ga-PSMA-11 injectable solutions according to Ph. Eur. Monograph that has recently been published, a trend to the instability of the standard PSMA-11, the same used as a precursor for [^68^Ga]Ga-PSMA-11 radiosynthesis, has been observed. This instability led to the formation of a side product in a time-dependent manner. The formation of this compound, besides making the implementation of the Ph. Eur. analytical method more difficult, negatively influenced the radiochemical yield and the radiochemical purity by increasing gallium-68 in colloidal and ionic forms.

**Results:**

The nature of the side product was investigated by adding chelators, such as EDTA, to PSMA-11 solutions and using the combination of UHPLC-HRMS. The results led to the definition of the side product structure, as ^nat^Fe-PSMA-11, from the combination of the high-affinity chelator HBED-CC, present in the molecule of PSMA-11, and environmental Fe (III).

**Conclusions:**

Strategies to reduce the risk of low radiolabeling yields and to increase the stability of the PSMA-11 in an aqueous solution were also discussed.

## Background

The radiopharmaceutical preparations or radiopharmaceuticals (RPs) are medicinal products that, when ready for use, contain one or more radionuclides included for a medical purpose (Gillings et al. 2020)*.*

Since radiopharmaceuticals are intended for human administration, they must undergo strict quality control tests before release for clinical use. Basically, quality control involves specific tests that ensure the purity, potency, product identity, biologic safety, and efficacy of radiopharmaceuticals (Saha 1992)*.*

The quality control (QC) of radiopharmaceuticals are described in the monographs of the European Pharmacopoeia (Ph. Eur.), including the related method of analysis with the experimental details and the acceptance criteria.

Ph. Eur. analytical methods must be verified in each laboratory, to make sure that the method has been implemented properly (i.e., system suitability test, detector linearity, and limit of quantification).

On the other hand, if a monograph for a radiopharmaceutical has not been published, as for the Investigational Medicinal Products (IMPs), or in case the monograph exists but for any reasons it is preferred to use a different method, the analytical methods must be validated before use in routine quality control procedures. The validation aims to prove that the methods are suitable for their intended purpose (Gillings et al. 2020)*.*

The Glu-urea-Lys (Ahx)-[(HBED-CC)] (PSMA-11) labelled with gallium-68, [^68^Ga]Ga-PSMA-11, is the radiopharmaceutical of choice for imaging of Prostate Cancer (PCa) (Fendler et al. 2017).

The Ph. Eur. Monograph “Gallium (^68^Ga) PSMA-11 injection” has just been published (Ph. Eur. Monograph “Gallium (68Ga)PSMA-11 Injection” 2021)*.*

While performing the QC of the [^68^Ga]Ga-PSMA-11 injectable solutions according to Ph. Eur. Monograph (Ph. Eur. Monograph “Gallium (68Ga) PSMA-11 injection” 2021)*,* we observed that the standard PSMA-11, the same used as a precursor for [^68^Ga]Ga-PSMA-11 radiosynthesis, was unstable in an (acidic) aqueous solution and a side product was formed in the solution, over time.

This instability caused a decrease in the PSMA-11 synthesis precursor concentration, which led to lower radiochemical yield (RCY), chemical purity (CP), and radiochemical purity (RCP) of the [^68^Ga]Ga-PSMA-11 preparations. Moreover, the PSMA-11 instability affected the implementation of the analytical method for the CP determination described in the Ph. Eur. Monograph “Gallium (^68^Ga) PSMA-11 injection” because of the impossibility of obtaining a long-term stable PSMA-11 *reference solution b*.

Since the stability of precursors and reagents are of utmost importance for developing a robust radiolabelling method that provides high and constant RCY and RCP, as well as for implementing Ph. Eur. analytical methods that are suitable, fast, and routinely reproducible, aims of this paper were to investigate the causes of PSMA-11 instability in an (acidic) aqueous solution and to identify the side product that was formed; to improve the stability of PSMA-11 in solution, and to evaluate possible strategies to limit the risk of a decrease in RCY, CP, and RCP of the [^68^Ga]Ga-PSMA-11 preparations caused by PSMA-11 instability.

## Materials and methods

### Chemicals and reagents

Reference standard PSMA-11, the same used as a precursor for [^68^Ga]Ga-PSMA-11 radiosynthesis, and the reference standard ^nat^Ga-PSMA-11, all are of GMP grade, was purchased from Advanced Biochemical Compounds, ABX (Radeberg, Germany). The lyophilized products were provided at − 20 ± 5 °C

Starting material, gallium-68 (t_1/2_ = 68 min, β + = 89%, and EC = 11%) for the [^68^Ga]Ga-PSMA-11 production, was routinely obtained as gallium-68 chloride ([^68^Ga]GaCl_3_) solution from a pharmaceutical grade germanium-68/gallium-68 generator (1.11 GBq, GalliaPharm®, Eckert&Ziegler Radiopharma GmbH, Berlin, Germany).

Disposable reagents (SCX reagent kit for the [^68^Ga]Ga-labeling of peptides) and disposable sterile cassettes (SCX fluidic kit for the [^68^Ga]Ga-labeling of peptides) for [^68^Ga]Ga-PSMA-11 radiosynthesis, all are of GMP grade, were purchased from Advanced Biochemical Compounds, ABX (Radeberg, Germany).

Strong cation exchange (SCX) cartridges Bond Elut-SCX (100 mg/3 mL, Agilent Technologies) were provided with the SCX reagent kit for the [^68^Ga]Ga-labeling of peptides.

SCX cartridges Strata® SCX (200 mg/3 mL) were purchased from Phenomenex (USA).

0.1 M sterile and ultrapure Hydrochloric Acid (HCl) for elution of germanium-68/gallium-68 generator was purchased from Eckert & Ziegler Radiopharma GmbH (Berlin, Germany).

Ethylenediaminetetraacetic Acid (EDTA), Ammonium Acetate, Methanol, and Acetonitrile (ACN) were purchased from Carlo Erba Reagents S.r.l. (Cornaredo, Milan, Italy); Trifluoroacetic Acid (TFA), Formic Acid (FA), and metal-free water (Fluka Water TraceSelect® for trace Analysis) were purchased from Merck Life Science S.r.l. (Milan, Italy); Ultrapure water (Milli-Q, 18.2 MΩ) was obtained from a Milli-Q® IQ Element purification (Merck KGaA, Darmstadt, Germany).

All chemicals were of analytical grade and were used without further purification. HPLC eluents (Milli-Q water, FA, ACN, and TFA) were of high-grade purity.

### Apparatus and chromatographic conditions

HPLC analysis was performed on a Thermo Scientific Dionex Ultimate 3000 HPLC system (Thermo Scientific, Bremen, Germany) equipped with LPG-3400SD pump, TCC-3000 column oven, UV VWD-3100 detector, and radiometric detector at NaI (Gabi Star, Elysia-Raytest, Germany) connected in series.

Reversed-Phase High-Performance Liquid Chromatography (RP-HPLC; ACE 3 μm C18, l = 0.6 m, Ø = 7 mm; Thermo Scientific, Bremen, Germany) with a linear A–B gradient (0–0.5 min 5% B, 0.5–10 min 5% B to 40% B, 10–11 min 40% B to 5% B, 11–16 min 5% B) at a flow rate of 0.6 mL/min and a total run time of 16 min was performed. Solvent A consisted of 0.1% TFA in Milli-Q water and solvent B of 0.1% TFA in ACN.

UV absorbance was measured at 280 nm. The column temperature was kept at 24 °C. The injection volume was 20 *μ*L.

The Chromeleon data system software (Version 7.2.8) was used for data acquisition and mathematical calculations.

Thin-layer Chromatography was performed using glass microfiber chromatography paper impregnated with silica-gel (iTLC-SG, Varian, Italy) developed in 1 M ammonium acetate/methanol (1:1) and analyzed using a single trace radio TLC-scanner (PET-miniGita, Elysia-Raytest, Straubenhardt, Germany) and evaluation software (Gina Star TLC, Elysia-Raytest, Straubenhardt, Germany).

### Reference solutions preparation

To implement the analytical method described in the Ph. Eur. Monograph “Gallium (^68^Ga) PSMA-11 injection” for CP and RCP determination of the [^68^Ga]Ga-PSMA-11 preparations, the ^nat^Ga-PSMA-11 *reference solution a*, and the PSMA-11 *reference solution b* were prepared according to Ph. Eur. Monograph.

The ^nat^Ga-PSMA-11 *reference solution a* was prepared by dissolving 50 *μ*g of anhydrous and trifluoroacetic acid-free ^nat^Ga-PSMA-11 in 1.0 mL of metal-free water.

The PSMA-11 *reference solution b* was prepared by dissolving 30 *μ*g of anhydrous and trifluoroacetic acid-free PSMA-11 in a solvent mixture of metal-free water and TFA (0.1% V/V) and diluting to V with the same solvent mixture (V being the maximum recommended dose in milliliters, which is 10 mL in our case) (Ph. Eur. Monograph “Gallium (68Ga) PSMA-11 injection” 2021)*.*

The reference solutions were stored at − 25 °C.

All the equipment for the reference solutions preparation was free from trace metal contamination.

### Analysis of PSMA-11 instability in (acidic) aqueous solution

During the storage of PSMA-11 *reference solution b* (3 *μ*g/mL) a side product was formed.

A series of analyses were performed to investigate if the side product formation was due to an impurity, an acidic degradation caused by TFA, or to the HBED-CC tendency of chelating metal ions already at room temperature.

**Fig. 1:**
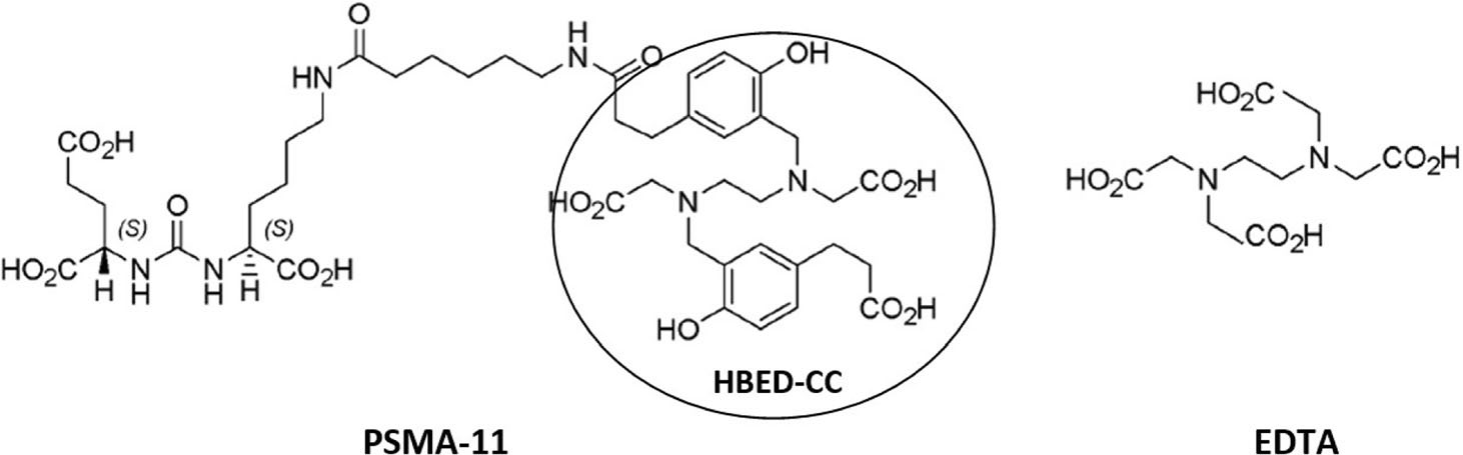
Chemical structure of PSMA-11 and EDTA

As it’s known, the structure of PSMA-11 is characterized by a highly efficient acyclic chelator with an EDTA-like structure and two additional phenol coordinating covalent bonds, the N, N′-bis [2-hydroxy-5-(carboxyethyl)benzyl] ethylenediamine-N, N′-diacetic acid (hereinafter HBED-CC, see Fig. 1), which demands rather low energy for metal ions complexing (Roesch and Riss 2010; Eder et al. 2014).

To exclude that the side product formation was due to an acidic degradation, the PSMA-11 *reference solution b* was prepared only with metal-free water and analyzed under the chromatographic conditions described before.

Since the obtained chromatograms showed the side product presence also in this solution during the storage at − 25 °C, the role of HBED-CC chelator in the side product formation was investigated. For this purpose, since HBED-CC is an acyclic chelator based on EDTA-type structure (see Fig. 1), a large excess of EDTA was added to a PSMA-11 *reference solution b*, prepared with metal-free water and TFA (0.1% V/V), 8 days old, showing the contaminant compound, with and without heating at 50 °C of the solution after adding EDTA.

The solutions were analyzed under the chromatographic conditions described before.

No interference due to EDTA was found out since EDTA absorbs at wavelengths between 222 and 236 nm (Sweetser and Bricker 1954; Dunstone and Payne 1959).

### Preparation of a long-term stable PSMA-11 *reference solution b*

The addition of EDTA allowed clarifying the role of likely metallic contaminants in the side product formation. Consequently, the PSMA-11 *reference solution b* (3 *μ*g/mL) was re-prepared by dissolving the anhydrous and trifluoroacetic acid-free PSMA-11 in a solution obtained by adding 100 mg of EDTA to 10 mL of a solvent mixture of metal-free water and TFA (0.1% V/V). Before adding the PSMA-11, the solution was incubated for one night at 50 °C to remove the metallic contaminants from the environment.

The obtained PSMA-11 *reference solution b* was stored at room temperature and analyzed 1 h after the preparation under the chromatographic conditions described before.

### UHPLC-HRMS analysis

To identify the chemical structure of the side product, a mass spectrometric study, using Ultra-High-Performance Liquid Chromatography–High-Resolution Mass Spectrometry (UHPLC–HRMS), of a 50 *μ*g/mL PSMA-11 standard solution, showing the contaminant compound, was performed.

Briefly, 5 *μ*L of the 50 *μ*g/mL PSMA-11 standard solution were injected into a Thermo Scientific Dionex Ultimate 3000 UHPLC coupled to a Thermo high-resolution Q Exactive mass spectrometer (Thermo Scientific, Bremen, Germany). The column (Zorbax SB-C18 RRHT, 2.1 × 50 mm, 1.8 μ particle size, Agilent Technologies), thermostated at 30 °C, was equilibrated with 0.3 mL/min of water 0.1% FA (A) with 5% ACN (B); after sample injection, B% was kept constant at 5% for 0.5′, then linearly increased from 5 to 40% in 9.5 min; B% was then brought to 5% in 1 min and kept at 5% B for 5 min for the reconditioning step. Each sample required a total run time of 16 min. Centroided MS and MS^2^ spectra were recorded in both positive and negative polarities from 300 to 1'800 and 200 to 2'000 m/z in Full MS/dd-MS^2^ (TOP2) mode, at a resolution of 70'000 and 17'500, respectively. The two most intense ions were selected for MS^2^ nitrogen-promoted collision-induced dissociation (NCE = 30). Precursor dynamic exclusion (15 s) and apex triggering (1 to 6 s) were set; peptide-like isotope pattern ions were preferred. The mass spectrometer was calibrated before the start of the analyses.

### [^68^Ga]Ga-PSMA-11 radiosynthesis

Radiosynthesis of [^68^Ga]Ga-PSMA-11 was carried out according to a well-established method (Dunstone and Payne 1959; Eder et al. 2012; Roesch 2013)*.*

[^68^Ga]Ga-PSMA-11 was prepared using 10 *μ*g of PSMA-11 precursor (10 *μ*L of a 1 *μ*g/*μ*L aqueous solution), loaded in an automated radiosynthesis module (GAIA V2™, Elysia-Raytest, Germany), by using disposable reagents assembled in a disposable sterile cassette.

The radionuclide gallium-68 was obtained as described in Chemicals and Reagents.

A series of modifications to the original radiosynthesis procedure was performed to investigate the possibility to reduce the risk of low radiolabeling yields due to the formation of side product in the aqueous solution of PSMA-11 synthesis precursor:

1. Using only freshly prepared aqueous solutions of PSMA-11 synthesis precursor (maximum 3 days from the preparation);
2. Replacing the Agilent SCX cartridge, included in the reagent kit (SCX reagent kit for the [^68^Ga]Ga- labeling of peptides) for the trapping and the purification of [^68^Ga]GaCl_3_ eluted (Dunstone and Payne 1959; Eder et al. 2012; Roesch 2013)*,* with a Phenomenex SCX cartridge that shows greater affinity for metal impurities, to minimize the presence in the reactor of metal ions from the [^68^Ga]GaCl_3_ eluate;
3. Increasing the amount of PSMA-11 precursor from 10 *μ*g aliquots to 30 *μ*g aliquots according to Ph. Eur. Monograph “Gallium (^68^Ga) PSMA-11 injection” (Ph. Eur. Monograph “Gallium (68Ga) PSMA-11 injection” 2021).

## Results

**Fig. 2:**
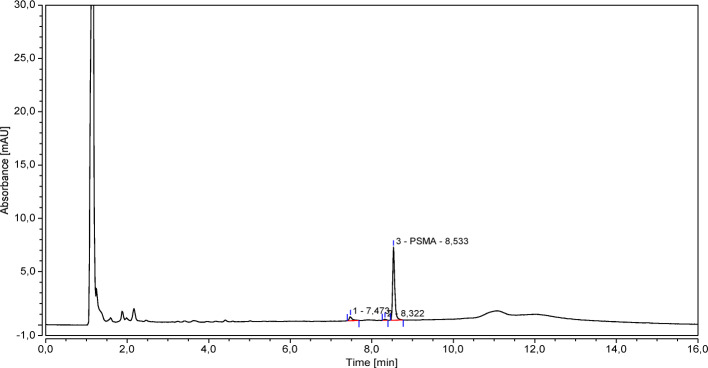
A typical chromatogram of a PSMA-11 *reference solution b* (3 *μ*g/mL) freshly prepared

Figure 2 shows a typical chromatogram of a PSMA-11 *reference solution b* (3 *μ*g/mL) freshly prepared.

The average Retention Time (RT) of the peak of PSMA-11 (hereinafter principal peak) is 8.5 min.

### Analysis of PSMA-11 instability in (acidic) aqueous solution

**Fig. 3:**
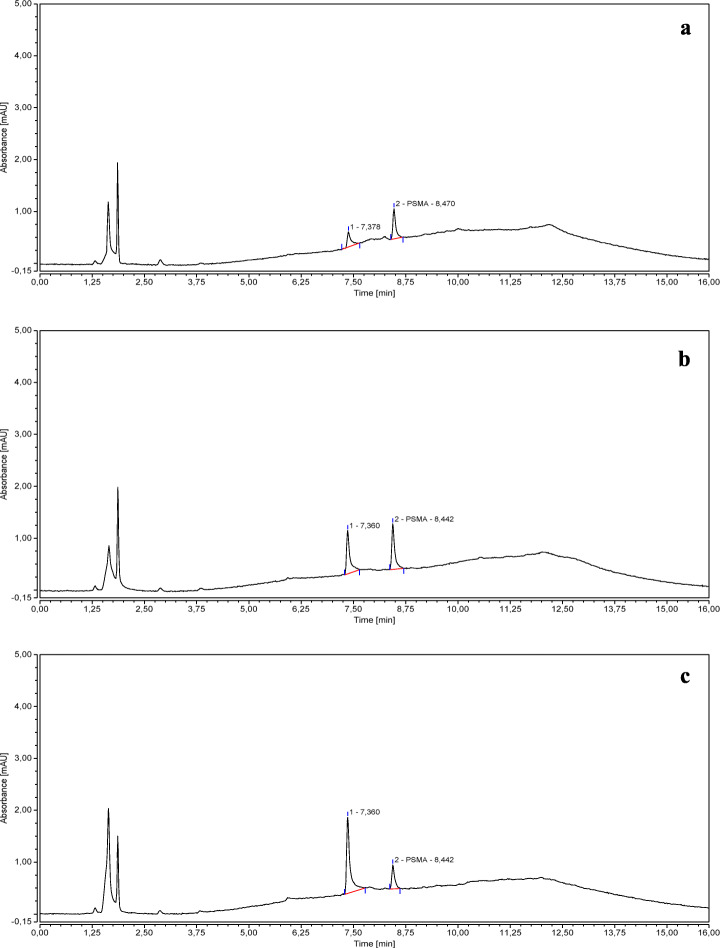
Chromatograms of a PSMA-11 *reference solution b* (3 *μ*g/mL*)* injected at 3 (**a**), 5 (**b**), and 8 (**c**) days from preparation

Figure 3 shows the chromatograms obtained from a PSMA-11 *reference solution b* (3 *μ*g/mL), injected at 3, 5, and 8 days from the preparation, and stored at − 25 °C.

The chromatograms clearly show that the PSMA-11 is not stable if dissolved in an acidic aqueous solution according to Ph. Eur. Monograph “Gallium (^68^Ga) PSMA-11 injection”.

When the PSMA-11 *reference solution b* (3 *μ*g/mL) was injected at 3 days from the preparation, a secondary peak with an average RT of 7.4 min was clearly detected in addition to the principal peak related to PSMA-11, whose area decreased over time in favor of the secondary peak area which became predominant (see Fig. 3). If we consider the sum of the side product peak area and the PSMA-11 peak area, the percentage of the side product increased from 0% to 67% in the time interval from 0 to 8 days.

In the chromatograms of the PSMA-11 *reference solution b* (3 *μ*g/mL*)* stored at room temperature, the secondary peak was clearly detectable already when injecting the solution at 6 h from the preparation, suggesting a combined time/temperature effect (data not shown).

To evaluate if the side product formation was also concentration-dependent, PSMA-11 standard solutions of different concentrations (i.e., 0.75, 1.5, 6, 12, and 50 μg/mL) were prepared by dissolving the anhydrous and trifluoroacetic acid-free PSMA-11 in a metal-free water and TFA (0.1% V/V) solvent mixture according to Ph. Eur. Monograph.

**Fig. 4:**
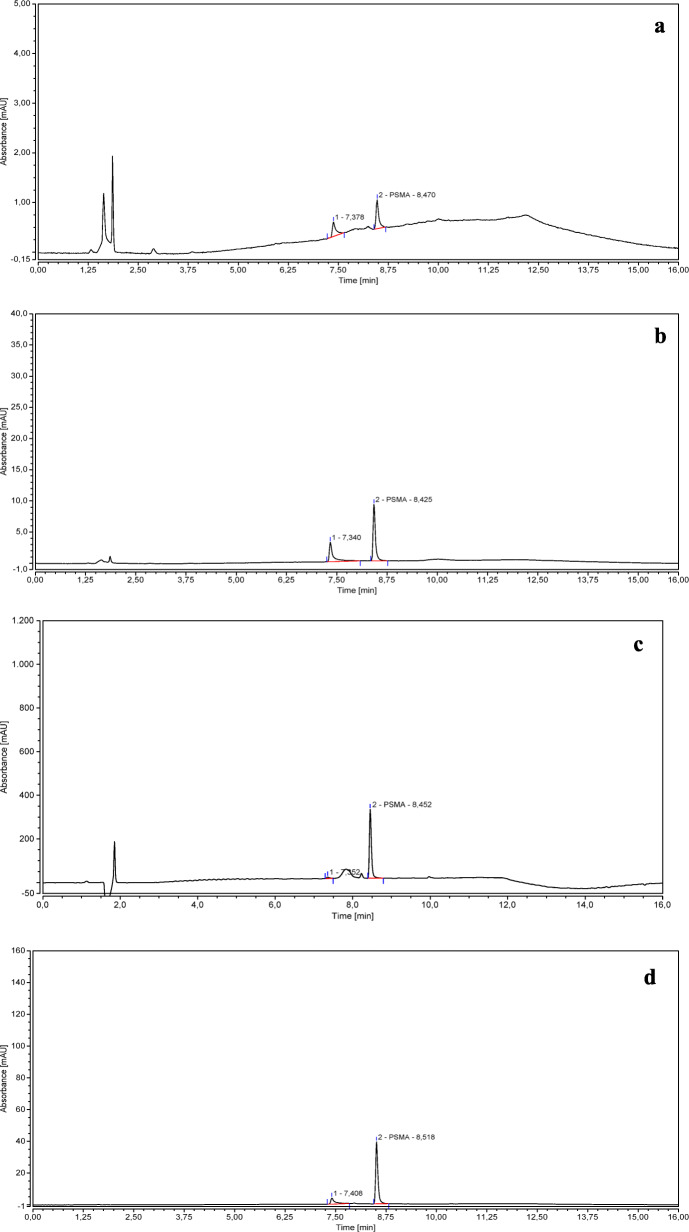
Chromatograms of 3 (**a**), 12 (**b**), and 50 (**c**) *μ*g/mL PSMA-11 standard solutions injected at 3 days from preparation and chromatogram of a 50 *μ*g/mL PSMA-11 standard solution injected 6 months after the preparation (**d**)

The secondary peak was detected in chromatograms obtained from all PSMA-11 solutions when they were injected at 3 days from the preparation, but it was more evident at low PSMA-11 concentration (3 *μ*g/mL) compared to high concentration (12 *μ*g/mL). In the chromatograms of the 50 *μ*g/mL PSMA-11 standard solution, this peak was detected only 6 months after the solution preparation (see Fig. 4).

The RT of the secondary peak was similar to the RT of PSMA-11 gallium-68 labelled peak (RT of [^68^Ga]Ga-PSMA-11 ~ 7.9 min, see Figs. 9, 10, and 11).

Interestingly, also the chromatograms of a PSMA-11 *reference solution b* (3 *μ*g/mL*)* prepared only with metal-free water clearly showed the secondary peak at 7.4 min RT, when the solution was injected at 3 days from the preparation (data not shown), allowing us to exclude the role of acidic degradation caused by TFA in the side product formation.

**Fig. 5:**
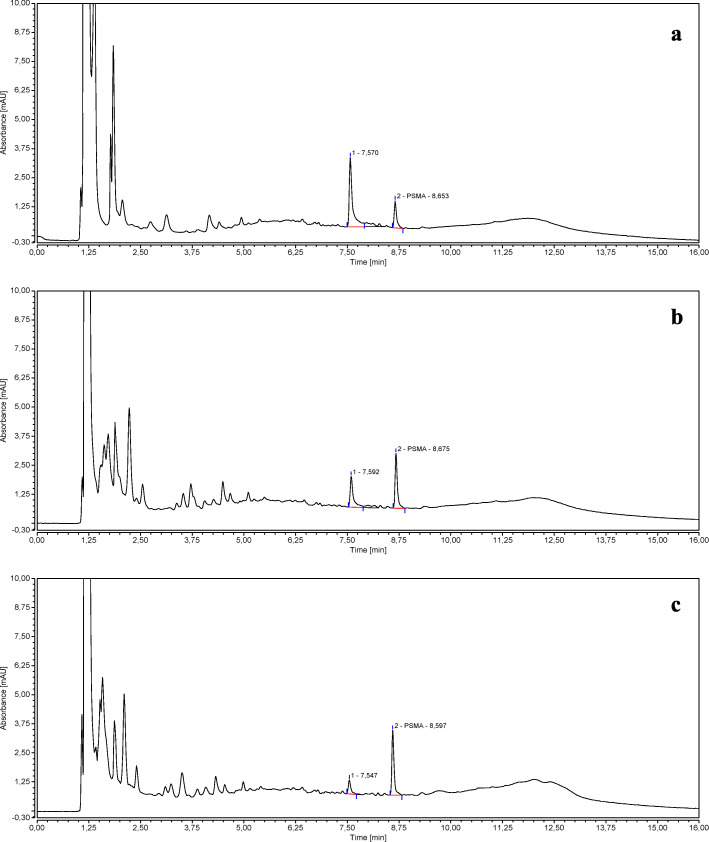
Chromatograms of a PSMA-11 *reference solution b* (3 *μ*g/mL) 8 days old, injected at 0.5 (**a**), 2 (**b**), and 5 (**c**) hours from adding EDTA and heated at 50 °C

Conversely, after adding EDTA to a PSMA-11 *reference solution b* 8 days old, showing the contaminant compound, with and without heating at 50 °C of the solution, the secondary peak area decreased over time in favor of the PSMA-11 peak area (see Fig. 5).

The complete transchelation effect of EDTA required 48 h without heating (data not shown) and 5 h when the PSMA-11 solution was heated to 50 °C after adding EDTA (see Fig. 5).

If we consider the sum of the side product peak area and the PSMA-11 peak area, the percentage of the side product decreased from c.a 70% to c.a 0.8% in both cases.

### Stability assessment of the PSMA-11 *reference solution b*

The experiments carried out with EDTA clarified the role of the interaction between HBED-CC and likely environmental metallic contaminants in the side product formation. Therefore, a long-term stable PSMA-11 *reference solution b* (3 *μ*g/mL) was prepared by adding EDTA (10 mg/mL) to the solvent mixture of metal-free water and TFA (0.1% V/V) recommended by the Ph. Eur. Monograph for dissolving the PSMA-11.

**Fig. 6:**
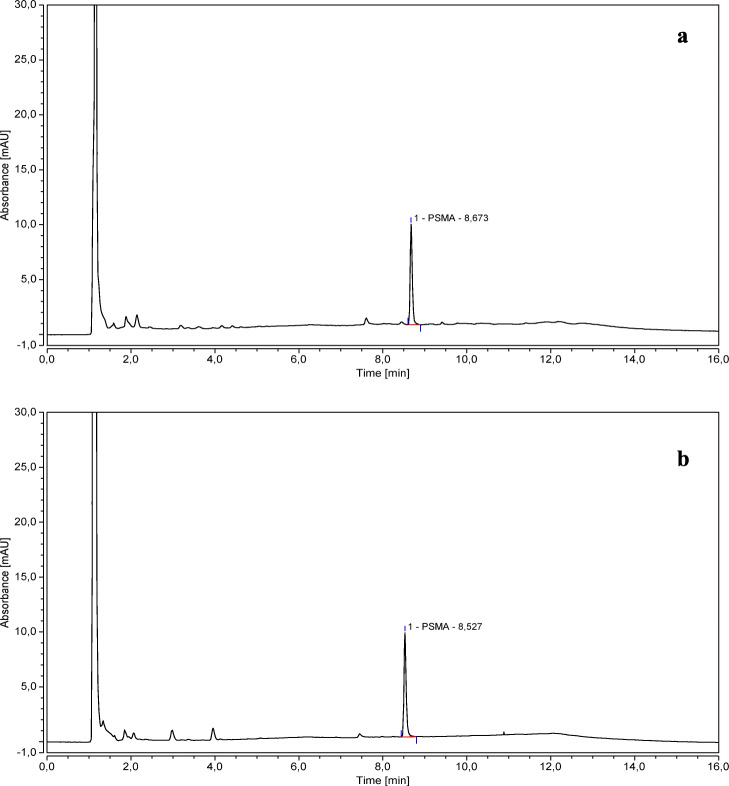
Chromatograms of a long-term stable PSMA-*11 reference solution b* (3 *μ*g/mL) freshly prepared (**a**) and at 12 months from preparation (**b**)

In chromatograms obtained from this solution, stored at room temperature, the secondary peak at 7.4 min RT was not detectable and the area of the peak due to PSMA-11 was stable at least for 12 months after the solution preparation (see Fig. 6).

### UHPLC-HRMS analysis

**Fig. 7:**
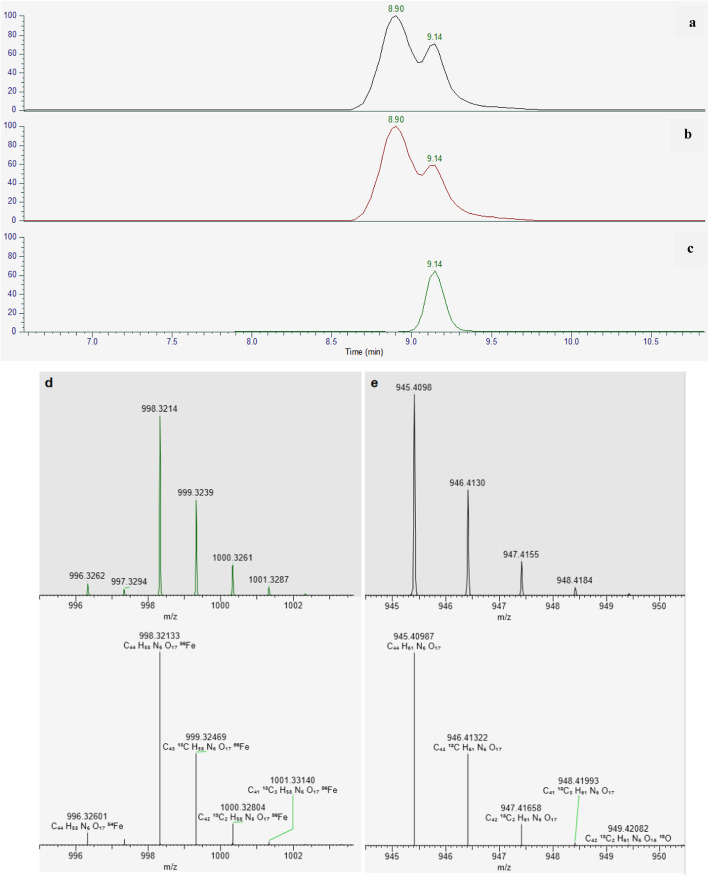
UHPLC-HRMS analysis of a 50 *μ*g/mL PSMA-11 standard solution

**Fig. 8:**
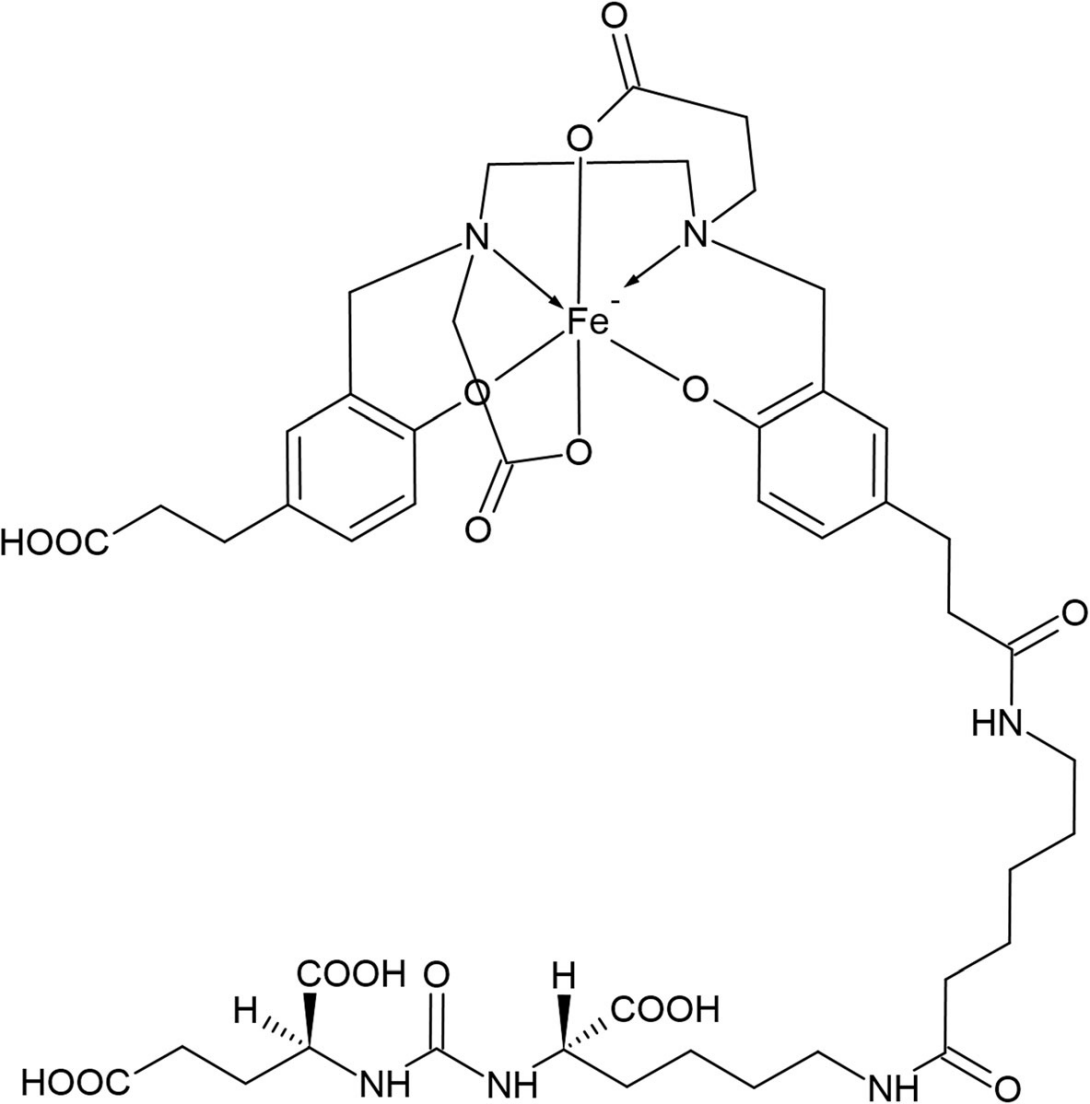
Chemical structure of Glu-urea-Lys (Ahx)-[Fe (HBED-CC)]^−^

The ESI MS negative base peak chromatogram of PSMA-11 showed two main peaks; the extracted ion chromatogram of the most intense ion of the peak at 8.9 min RT (998.3214 m/z) shows 2 not resolved peaks which can be attributed to the [C_44_H_58_FeN_6_O_17_] ^-^ ion, which corresponds both in terms of mass and isotopes patterns/relative intensity (the bottom right frame of Fig. 7) to a complex of PSMA-11 with Fe (III) (structure reported in Fig. 8). The not resolved peak at 8.9 could be reasonably attributed to the two different diastereomeric forms of the complex. The extracted ion chromatogram of the most intense ion of the peak at 9.14 min RT (945.4099 m/z) shows a peak which can be attributed to the [M-H]- ion of HBED-CC; as shown in the bottom left frame of Fig. 7, the experimental (top) and predicted (bottom) spectra correspond both in terms of mass and isotopes patterns/relative intensity.

### Analysis of [^68^Ga]Ga-PSMA-11 preparations

We observed that the side product formation in the aqueous solution of PSMA-11 synthesis precursor can cause an increase in the percentage of gallium-68 in colloidal (i.e., 1.2% vs 5%) and ionic (i.e., 0.4% vs 10%) forms in the final product and, consequently, a decrease in the percentage of radiolabeling yield non-decay corrected (n.d.c.) at end of synthesis (EOS) (i.e., 91% vs 43%), as well as in the percentage of RCP of the [^68^Ga]Ga-PSMA-11 preparations.

**Fig. 9:**
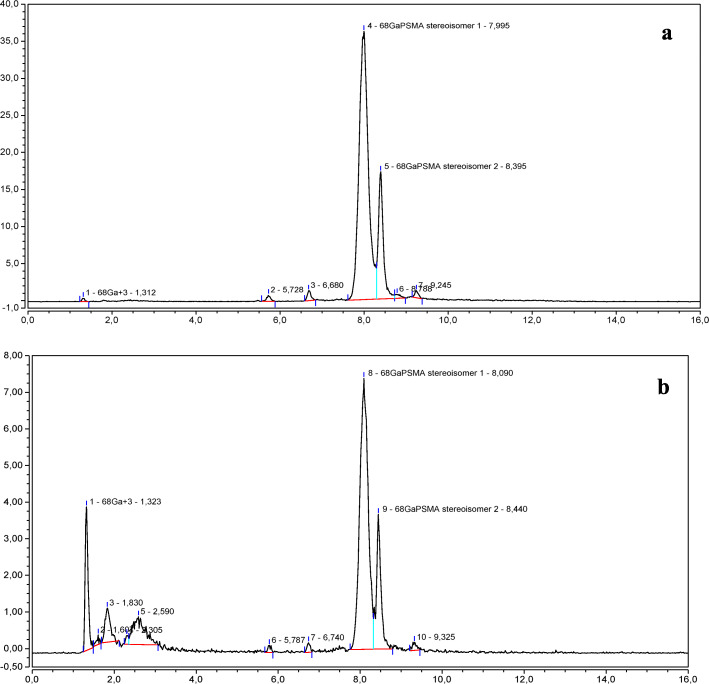
Radiochromatograms of [^68^Ga]Ga-PSMA-11 solutions prepared by gallium-68 radiolabeling a freshly prepared PSMA-11 aqueous solution (**a**) and a PSMA-11 aqueous solution 8 days old (**b**)

In radiochromatograms obtained by analysis of a [^68^Ga]Ga-PSMA-11 solution prepared from 10 *μ*L of an aqueous solution (1 *μ*g/*μ*L) of PSMA-11 synthesis precursor 8 days old (see Fig. 9b), the area of the peak due to gallium-68 in ionic form was greater (up to 10 times) than in those obtained by using the same aliquot of a freshly prepared (maximum 3 days old) PSMA-11 synthesis precursor solution (see Fig. 9a).

**Fig. 10:**
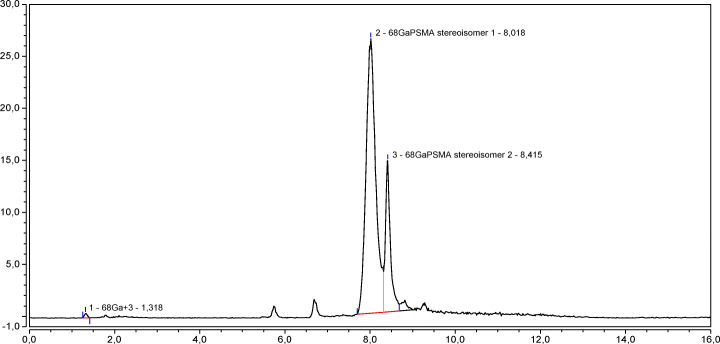
Radiochromatogram of a [^68^Ga]Ga-PSMA-11 solution prepared from 30 *μ*g of PSMA-11 precursor

**Fig. 11:**
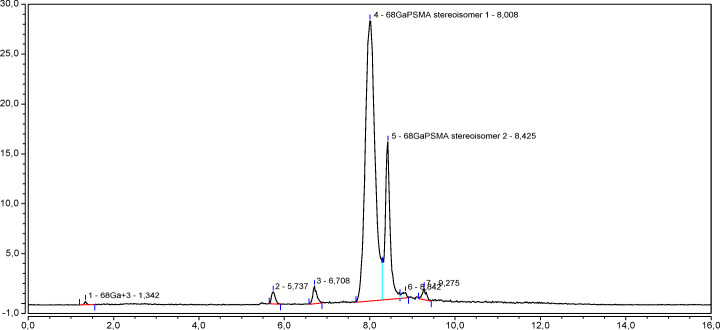
Radiochromatogram of a [^68^Ga]Ga-PSMA-11 solution prepared with a Phenomenex SCX cartridge

Conversely, in radiochromatograms obtained 1) by using a synthesis precursor solution (1 *μ*g/*μ*L) 15 days old and increasing the PSMA-11 precursor in the radiosynthesis from 10 to 30 *μ*L (see Fig. 10), or 2) by performing the radiosynthesis with 10 *μ*L of a not freshly prepared (≥ 3 days old) precursor solution (1 *μ*g/*μ*L) and using a Phenomenex SCX cartridge more suitable to retain metal impurities than Agilent SCX cartridge (see Fig. 11), the area of the peak due to gallium-68 in ionic form was more comparable to the one obtained by performing the radiosynthesis with 10 *μ*L of a freshly prepared precursor solution (see Fig. 9a).

TLC analysis of gallium-68 in the colloidal form provided comparable data.

## Discussion

While performing the QC of the [^68^Ga]Ga-PSMA-11 injectable solutions according to Ph. Eur. Monograph “Gallium (^68^Ga) PSMA-11 injection”*,* we observed that PSMA-11 *reference solution b* (3 *μ*g/mL) required for the CP determination of the [^68^Ga]Ga-PSMA-11 preparations (Ph. Eur. Monograph “Gallium (68Ga) PSMA-11 injection” 2021), was unstable in an acidic aqueous solution. This instability led to the formation of a side product, with a peak at 7.4 min RT, when the solution was stored at − 25 °C already after 3 days from preparation.

The same phenomenon occurred in the PSMA-11 aqueous solution (1 *μ*g/*μ*L) usually used for the [^68^Ga]Ga-PSMA-11 radiosynthesis.

The side product was observed to be more detectable when working at lower concentrations (see Fig. 4).

Since the stability of the precursor is of utmost importance in the synthesis of the radiopharmaceuticals because of its impact on RCY and RCP, a series of analyses were performed to determine the cause of instability.

The obtained results led us to hypothesize that the side product was the result of the time concentration-dependent interaction, already at room temperature, between HBED-CC and metal ions probably present in the PSMA-11 aqueous solutions both with and without TFA, because of HBED-CC fast coordination kinetic (Spang et al. 2016). Therefore, to prepare the PSMA-11 *reference solution b,* EDTA (10 mg/mL) was added to the solvent mixture of metal-free water and TFA (0.1% V/V) recommended by the Ph. Eur. Monograph for dissolving the PSMA-11. In order to remove the metallic contamination from the solvents, the solvent mixture of EDTA, TFA, and metal-free water was incubated for one night at 50 °C before adding the PSMA-11. Nevertheless, we observed that to obtain a stable *reference solution b,* it was also necessary to incubate the solution for 1 h at room temperature after adding the PSMA-11.

Therefore, metallic contamination coming from the equipment and solvents used for the standard PSMA-11 synthesis cannot be ruled out in the side product formation.

To identify the metal ion responsible for the formation of the side product, the UHPLC–HRMS of a 50 *μ*g/mL PSMA-11 standard solution, showing the contaminant compound, was performed.

The UHPLC–HRMS showed that the side product is likely a complex between HBED-CC and Fe (III) with molecular formula: [C_44_H_58_FeN_6_O_17_]^−^ and structure reported in Fig. 8 (Eder et al. 2008)*.*

This observation could be justified by the fact that Fe (III) is a ubiquitous metal (Cusnir et al. 2017)*.*

Moreover, it could justify the large excess of EDTA required for obtaining the transchelation effect of EDTA on HBED-CC metal ions complex, because the EDTA binds Fe (III) with a lower affinity (log K_EDTA-Fe_ = 25.1) than the HBED-CC (log K_HBED-CC-Fe_ = 36.74) (Harris 2015; Motekaitis et al. 1991; Imberti et al. 2019)*.*

The very high affinity between HBED-CC and Fe (III) would also justify the presence in the chromatograms of the secondary peak already at 6 h from the preparation of solutions when they are stored at room temperature, as well as the relatively slow formation of the side product (within 3 days from the preparation of solutions) in the PSMA-11 solutions stored at − 25 °C, temperature at which the kinetic of coordination is negligible. This behavior would be less probable in presence of a metal ion with a lower affinity for the HBED-CC as Cu (II) (log K_HBED-CC-Cu_ = 23.40) (Ma et al. 1994; Long 1990)*.*

Finally, the formation of the complex between HBED-CC and Fe (III) in the aqueous solutions of PSMA-11 synthesis precursor could also justify the decrease in the radiolabeling yield by using not freshly prepared solutions or solutions more than 3 days old, because the affinity of HBED-CC for both Ga (III) and Fe (III) is similarly high (log K_*Ga*_ = 37.73, log K_*Fe*_ = 36.74) (Imberti et al. 2019; Wadas et al. 2010), and higher than the affinity between HBED-CC and other commonly ubiquitous metal ions Cu (II) or Zn (II) or Co (II) or Ni (II) (Ma et al. 1994; Wadas et al. 2010)*.*

The formation of the ^nat^Fe-PSMA-11 complex would also explain why the peak due to side product has an RT more similar to the RT of [^68^Ga]Ga-PSMA-11 peak than the RT of “cold” PSMA-11 peak.

Since the Fe (III) can compete with the [^68^Ga]Ga (III) for binding the HBED-CC, and the [^68^Ga]GaCl_3_ concentration in generator eluates is very low (Imberti et al. 2019)*,* to reduce the risk of low radiolabeling yields it was necessary to increase the amount of PSMA-11 precursor from 10 *μ*g to 30 *μ*g or to use an SCX cartridge which shows greater affinity for metal impurities in order to minimize the presence of metal ions from the [^68^Ga]GaCl_3_ eluted in the reactor.

These results on PSMA-11 instability are not in agreement with data previously reported by Migliari et al. (Migliari et al. 2017)*.* These authors validated an analytical HPLC method to analyze the [^68^Ga]Ga-PSMA-11, but they attributed the not resolved peak, detected in HPLC trace of the PSMA-11, to two different diastereomeric forms of PSMA-11. We demonstrated that this not resolved peak should be attributed to coordination between PSMA-11 and a ubiquitous metal ion.

Moreover, we observed that performing the analysis of the PSMA-11 solutions at UV absorbance (*λ*) of 220 nm, according to report in the paper of Migliari et al. (Migliari et al. 2017), as well as in the Chemistry, Manufacturing, and Controls (CMC) of the PSMA-11 supplied by ABX, the secondary peak is much less detectable than performing the analysis at 280 nm, as reported in the Ph. Eur. Monograph.

Also, a common practice, consisting in the analysis of a PSMA-11 *reference solution b* and ^nat^Ga-PSMA-11 *reference solution a* mixture to perform the system suitability test of the chromatographic system before QC starting, doesn’t allow the detection of the secondary peak at 7.4 min RT because of its co-elution with the ^nat^Ga-PSMA-11 stereoisomer 1 peak.

## Conclusions

The stability of the PSMA-11 in an (acidic or not acidic) aqueous solution is poor and, over time, a side product is formed (RT ~ 7.4 min. See Fig. 3), which is more evident at low concentrations.

We have demonstrated that it’s due to the interaction between HBED-CC and Fe (III), a ubiquitous metal ion, possibly also in the environment of the standard PSMA-11 synthesis.

Since, the HBED-CC binds both Ga (III) and Fe (III) with a very high affinity, the formation of the complex between HBED-CC and Fe (III) in PSMA-11 aqueous solutions has an important impact on the [^68^Ga]Ga-PSMA-11 radiosynthesis because it could increase gallium-68 in colloidal and ionic forms in the final product and, consequently, decrease radiochemical yield, chemical and radiochemical purity of the radiopharmaceutical preparations.

In this paper are presented possible strategies to reduce the risk of low radiolabeling yields, as well as of a low CP and RCP of the [^68^Ga]Ga-PSMA-11 preparations.

Moreover, this paper describes a possible way of obtaining a long-term stable PSMA-11 *reference solution b,* which is required by the Ph. Eur. Monograph “Gallium (^68^Ga) PSMA-11 injection” for performing the CP determination of the [^68^Ga]Ga-PSMA-11 injectable solutions.

Finally, this study highlights how the availability of a chelator with a preference for Ga (III) over other likely metal contaminants in eluates or used equipment would be very important not only to obtain radiotracers with higher molar activity but also for developing a suitable, fast and routinely reproducible analytical method for the QC.

## Data Availability

All data generated or analysed during this study are included in this published article. More data are available from the corresponding author on request.

## Abbreviations

ACN: Acetonitrile
CP: Chemical Purity
EDTA: EthyleneDiamine Tetraacetic Acid
EOS: End Of Synthesis
ESI: Electrospray Ionization
EU: European Union
HCl: Hydrochloric acid
HPLC: High-Performance Liquid Chromatography
IMPs: Investigational Medicinal Products
MS: Mass Spectrometry
NDC: Non-Decay Corrected
Ph. Eur.: European Pharmacopoeia
PCa: Prostate Cancer
PSMA: Prostate-Specific Membrane Antigen
QC: Quality Control
RCP: RadioChemical Purity
RCY: Radio Chemical Yield
RP-HPLC: Reversed-Phase High-Performance Liquid Chromatography
RPs: Radiopharmaceutical Preparations or Radiopharmaceuticals
RT: Retention Time
SCX: Strong Cation Exchange
TFA: Trifluoroacetic Acid
UHPLC–HRMS: Ultra-High-Performance Liquid Chromatography–High-Resolution Mass Spectrometry
V: Volume
